# Influence of Maternal Protein Restriction in Primiparous Beef Heifers during Mid- and/or Late-Gestation on Progeny Feedlot Performance and Carcass Characteristics

**DOI:** 10.3390/ani12050588

**Published:** 2022-02-25

**Authors:** Janna J. Block, Megan J. Webb, Keith R. Underwood, Michael G. Gonda, Adele A. Harty, Robin R. Salverson, Rick N. Funston, Kenneth C. Olson, Amanda D. Blair

**Affiliations:** 1 Hettinger Research Extension Center, North Dakota State University, Hettinger, ND 58639, USA; 2 Department of Animal Science, South Dakota State University, Brookings, SD 57007, USA; megan.webb@easternwv.edu (M.J.W.); keith.underwood@sdstate.edu (K.R.U.); michael.gonda@sdstate.edu (M.G.G.); adele.harty@sdstate.edu (A.A.H.); robin.salverson@sdstate.edu (R.R.S.); kenneth.olson@sdstate.edu (K.C.O.); amanda.blair@sdstate.edu (A.D.B.); 3 West Central Research & Extension Center, University of Nebraska-Lincoln, North Platte, NE 69101, USA; rick.funston@unl.edu

**Keywords:** beef, carcass, feedlot performance, fetal programming, maternal nutrient restriction, metabolizable protein

## Abstract

**Simple Summary:**

Maternal nutrient restriction in beef cows can impact developmental processes in the fetus, which may influence lifetime performance. If feed is limited, a maternal deficiency in protein and/or energy can occur. As a result, the fetus may receive inadequate levels of nutrients, potentially altering fetal development. In the present study, we evaluated the impact of a metabolizable protein restriction during mid- and/or late-gestation on progeny carcass characteristics. First-calf heifers were provided with a diet that either met their metabolizable protein requirements or caused an 80% restriction in metabolizable protein during mid- and/or late-gestation in a crossover design. Our results suggest that heifers catabolized lean body tissue, but not body fat stores, to overcome the metabolizable protein deficiency. However, restricting metabolizable protein in gestating heifers did not substantially influence the feedlot performance or carcass characteristics of their offspring. The restricted dams may have buffered their offspring from the metabolizable protein limitation during gestation.

**Abstract:**

This study investigated the impacts of metabolizable protein (MP) restriction in primiparous heifers during mid- and/or late-gestation on progeny performance and carcass characteristics. Heifers were allocated to 12 pens in a randomized complete block design. The factorial treatment structure included two stages of gestation (mid- and late-) and two levels of dietary protein (control (CON); ~101% of MP requirements and restricted (RES); ~80% of MP requirements). Half of the pens on each treatment were randomly reassigned to the other treatment at the end of mid-gestation. Progeny were finished in a GrowSafe feeding system and carcass measurements were collected. Gestation treatment x time interactions indicated that MP restriction negatively influenced heifer body weight (BW), body condition score, and longissimus muscle (LM) area (*p* < 0.05), but not fat thickness (*p* > 0.05). Treatment did not affect the feeding period, initial or final BW, dry matter intake, or average daily gain of progeny (*p* > 0.05). The progeny of dams on the RES treatment in late gestation had a greater LM area (*p* = 0.04), but not when adjusted on a hot carcass weight basis (*p* > 0.10). Minimal differences in the animal performance and carcass characteristics suggest that the level of MP restriction imposed during mid- and late-gestation in this study did not have a significant developmental programming effect.

## 1. Introduction

The nutrient status of gestating beef cows can have various long-term implications on the growth, feed intake and efficiency, and performance of offspring [1]. As mature mass and body composition can be altered by starvation or protein deficiency early in fetal life [2], progeny born to dams that were nutritionally restricted during gestation may have reduced skeletal muscle development and the efficiency of nutrient utilization. Nutrient restriction during gestation in livestock may result in unfavorable fetal and postnatal growth, nutrient utilization and health, as well as changes in body composition and meat quality [3]. Skeletal muscle development begins at an early stage of embryonic development, with primary muscle fibers in cattle estimated to begin forming at less than 47 days of fetal life and secondary muscle fibers around 90 days of fetal life [4]. Skeletal muscle is particularly susceptible to maternal nutrient deficiency due to its reduced priority in nutrient partitioning compared with other organs during development and the fact that muscle fiber numbers do not increase after birth in ruminants [5,6]. This is significant because any challenges or restrictions that compromise muscle development in utero could result in reduced muscle mass throughout the animal’s lifetime. Myocytes, adipocytes, and fibroblasts are all derived from mesenchymal stem cells early in fetal development, and evidence suggests that factors such as stress and maternal undernutrition can shift cell differentiation away from muscle development and result in the replacement of muscle fibers with adipocytes [7].

In many forage-based production systems, protein may be deficient in gestating beef cow diets due to increasing nutrient requirements, forage quality issues, environmental conditions and/or inadequate supplementation [8]. While several studies, such as Stalker et al. [9] and Summers et al. [10], have considered the effect of level of crude protein (CP) in late gestation, dietary CP can be extensively modified during ruminal fermentation and does not directly translate to protein supplied to the ruminant animal. Metabolizable protein (MP) is true protein absorbed in the intestine, consisting of microbial protein and ruminally undegraded protein sources (RUP). Although MP has been utilized for over 20 years to define protein requirements of beef cattle [11], limited data (e.g., [12,13,14,15,16]) are available on the effect of a MP restriction during gestation and the subsequent developmental programming effect. 

To develop a more comprehensive understanding of the potential influence of a gestational MP restriction, our hypothesis was that MP restriction in mid- and/or late-gestation would result in decreased dam performance, along with reductions in calf postnatal growth, reduced feed efficiency and increased carcass adiposity. Therefore, the objectives of this study were to investigate the impacts of MP restriction in mid- and/or late-gestation on the performance of dams and feedlot performance and carcass characteristics of progeny. 

## 2. Materials and Methods

### 2.1. Animals and Experimental Design

The South Dakota State University (SDSU) Institutional Animal Care and Use Committee approved all procedures involving animals (approval number 13-076A). A detailed description of the animals, experimental design, and experimental treatments is available in a companion paper by Webb et al. [17]. In brief, pregnant two-year-old Angus × Simmental heifers (*n* = 108) were pen-fed at the SDSU Cottonwood Range and Livestock Field Station during the treatment period. Treatments were arranged in a 2 × 2 factorial structure with 2 levels of dietary MP provided during 2 stages of gestation (mid and late) in a randomized complete block design. The mid-gestation treatment was applied from mean day 148 through 216 of gestation, while the late-gestation treatment was applied from mean day 217 of gestation through parturition). Dietary MP levels included: control (CON; approximately 101% of MP requirement) and restricted (RES; approximately 80% of MP requirement supplied) based on Level 2 of NRC [11]. 

At the end of the mid-gestation treatment period, half of the pens on the CON treatment were crossed over to the RES treatment and half of the pens on the RES treatment were crossed over to the CON treatment, with the other half of the pens remaining on the same treatment in a Balaam’s Design [18] to evaluate carryover effects from mid- to late gestation. This experimental design resulted in 4 treatment combinations (CON-CON, CON-RES, RES-CON, and RES-RES). Each treatment combination was randomly assigned to one pen per block for a total of three pen replicates per treatment combination. Each pen contained from 8 to 10 heifers. 

Heifer performance data were collected at trial initiation, at treatment crossover, and approximately 3 weeks prior to calving. Individual heifer body weight (BW) was recorded, and body condition score (BCS) was determined using a 9-point scale (1 = extremely emaciated to 9 = extremely obese; according to Wagner et al., [19]) with observations from the same 3 trained, independent observers at each timepoint. Mean BW and BCS of heifers at initiation of the experiment were 437 ± 17.2 kg and 5.25 ± 0.147, respectively. Ultrasound images were recorded and analyzed by a trained, certified technician to determine 12th rib subcutaneous fat thickness and longissimus muscle (LM) area for each heifer using an Aloka 500 V (Aloka, Wallingford, CT, USA). Mean fat thickness and LM area of heifers at initiation of the experiment were 0.54 ± 0.012 cm and 48.9 ± 2.32 cm^2^, respectively. 

Heifers were removed from their respective pens and dietary treatments immediately prior to or following calving. Within 24 h of birth, calves were weighed and tagged, and male calves were castrated via banding using a premium castration ring plier (Neogen Corp., Lansing, MI, USA). Cow–calf pairs were managed as a common group on native pastures through weaning, with no further nutritional restrictions imposed on dams or their offspring. Five calves were removed from the study prior to weaning due to death or issues with their dam that inhibited study protocols and objectives. 

### 2.2. Progeny Weaning and Feedlot Management 

Steer and heifer progeny (*n* = 103) were weaned at an average age of 196 ± 15 days, with the weaning date defined as day 0 of the feeding period. After backgrounding on high-quality grass hay and corn-based dried distiller grains for two weeks at the SDSU Cottonwood Range and Livestock Field Station, progeny were shipped approximately 430 km to the University of Nebraska-Lincoln West Central Research and Extension Center in North Platte, NE, USA. They were allocated to four feedlot pens based on sex and method of conception (artificial insemination (AI) or natural service) and adapted to a final finishing diet over 110 days. Progeny remained within these four groups and were placed in a GrowSafe™ feeding system (GrowSafe Systems Ltd., Airdrie, AB, Canada) for a 10-day adaptation period. Initial BW was recorded on two consecutive days following the adaptation period and used to determine average daily gain (ADG), and gain-to-feed ratio (G:F). Individual feed intake data collection was initiated on day 47 for AI-bred calves and day 68 of the feeding period for bull-bred calves. All progeny received the same diet throughout the feeding period. The finishing ration consisted of 48% dry rolled corn, 7% grass hay, 40% corn gluten feed, and 5% protein supplement (dry matter (DM) basis). The protein supplement was formulated to provide minerals and vitamins to meet nutrient requirements [11] using ground corn, limestone, iodized salt, ammonium chloride, trace mineral mix, vitamins A, D, and E, monensin (Rumensin, Elanco Animal Health, Greenfield, IN, USA), and tylosin phosphate (Tylan 40, Elanco Animal Health, Greenfield, IN, USA). Nutrient composition for the finishing ration was evaluated by wet chemistry analysis (Ward Laboratories, Inc., Kearney, NE, USA). The ration contained 75.05% dry matter, 11.47% CP, 1.79 Mcal/kg net energy for maintenance (NE_m_), and 1.61 Mcal/kg net energy for gain (NE_g_) on a DM basis. All steers received an initial feedlot implant of Revalor-IS (80 mg trenbolone acetate and 16 mg estradiol) and heifers received Revalor-IH (80 mg trenbolone acetate and 8 mg estradiol; Merck Animal Health, Madison, NJ, USA) on day 45 of the feeding period. Cattle were re-implanted with Revalor-200 (200 mg trenbolone acetate and 20 mg estradiol; Merck Animal Health, Madison, NJ, USA) and dewormed with Agrimectin (Agri Laboratories Ltd., St. Joseph, MO, USA) on day 148 of the feeding period. 

### 2.3. Progeny Harvest and Carcass Evaluation

Progeny were fed and managed to maintain health and achieve an industry average endpoint of approximately 1.3 cm of backfat at harvest. The AI-bred steers and heifers were shipped approximately 100 km to Tyson Fresh Meats in Lexington, NE on day 219 of the feeding period, and bull-bred steers and heifers were shipped to the same processing facility on day 240. Individual carcass measurements included hot carcass weight (HCW), LM area, 12th rib fat thickness, and estimated percentage of kidney, pelvic and heart fat (KPH) to calculate yield grade. Marbling score was also evaluated. Yield grade and marbling score were evaluated according to the United States Standards for Grades of Carcass Beef [20]. Final live BW was determined as HCW divided by 0.625. 

### 2.4. Statistical Analysis

All dam performance, progeny feedlot performance and carcass data were analyzed using dam pen assignments as the experimental unit. Period was included as a repeated measure for variables that were measured more than once, including BW, BCS, and ultrasound measurements. Variance–covariance structures were evaluated for each variable using repeated measures analyses, and the structure that best fit the data was selected based on the Schwarz’s Bayesian Information Criteria (BIC). For repeated variables (BW, BCS, and ultrasound measurements), initial measures were included in the model as covariates. Repeated-measure variables were also analyzed as the change in each variable during each period of gestation (e.g., end BW—beginning BW). Calf sex was included as a fixed effect for calf data. Dam performance and calf initial and final BW, and feedlot performance measures including dry-matter intake (DMI), ADG, and G:F, and carcass characteristics (HCW, LM area, 12th rib fat thickness, KPH, yield grade, and marbling score) were analyzed as a factorial treatment structure in a randomized complete block appropriate for a Balaam’s Design [18] for crossover experiments using the MIXED procedure of SAS (SAS Institute, Cary, NC, USA). Fixed effects included mid- and late-gestation treatment’s main effects and their interaction. The denominator degrees of freedom were approximated using the Kenward–Roger option in the model statement [21] for all analyses. Least squares means and SEM were estimated and separated by protected LSD (i.e., the PDIFF option). The influence of maternal nutritional treatments on the proportion of cattle assigned to each USDA Yield and Quality Grade were analyzed using a binary distribution in the GLIMMIX procedure of SAS, using the model described above. The least-squares means and SEM of the proportions were estimated using the ILINK option and separated as described above. All tests were considered significant at *p* ≤ 0.05, with tendencies considered at *p* < 0.10.

## 3. Results

### 3.1. Dam Performance

Dam performance responses (Table 1 and Table 2) provide context for progeny results. A mid-gestation treatment (CON vs. RES) × time (treatment crossover and end of study) interaction was observed (*p* < 0.05; Table 1) for changes in heifer BW, BCS, and LM area.

No differences (*p* > 0.05) were detected for change in 12th rib fat thickness due to the main effect of treatment or any treatment × period of gestation interaction. Restricting MP caused a greater reduction (*p* < 0.05) in BW and LM area and tended (*p <* 0.10) to cause a greater reduction in BCS during the mid-gestation feeding period. No carryover effect of mid-gestation treatment on BW, BCS, or LM area change from treatment crossover to the end of the study was detected. Late-gestation treatment (CON vs. RES) × time interactions were also observed for changes in heifer BW, BCS, and LM area (*p* < 0.05; Table 2). Consistent with responses in mid-gestation, 12th rib fat thickness did not respond to treatments in late gestation (*p* > 0.05). All heifers gained BW during the late gestation period; however, the interaction for BW change indicated the MP restriction resulted in lower BW gains (*p =* 0.001). In addition, restricted heifers lost BCS in the late-gestation period whereas heifers on the CON treatment maintained BCS (*p =* 0.007). These responses suggest that heifers catabolized lean body tissue, but not body fat stores, to overcome the MP deficiency.

### 3.2. Progeny Performance

No interactions (*p* > 0.10) were observed for mid- × late-treatment for any calf response variables; therefore, only the main effect means are presented in Table 3 and Table 4. Nutritional treatments offered to heifers during mid- and/or late-gestation did not affect subsequent calving difficulty or calf vigor (*p* > 0.10). Calf birth and weaning weights were not affected (*p* > 0.10; mean 30 ± 1.82 and 208 ± 8.96 kg, respectively) by mid- or late-gestation treatment. No differences (*p* > 0.05) were observed in initial or final BW, DMI, or ADG of progeny due to maternal nutritional treatment during the backgrounding and finishing phase. Progeny subjected to the late-gestation MP restriction tended to have improved G:F (*p* = 0.08).

### 3.3. Progeny Carcass Characteristics

No influence (*p* > 0.10; Table 4) of maternal diet was observed during gestation for progeny HCW, 12th rib fat thickness, KPH, yield grade, or marbling score. Longissimus muscle area for calves whose dams were restricted in late gestation was greater (*p* = 0.039) compared with those from dams on the control treatment; however, there was no difference among treatment groups (*p* = 0.231) when LM area was analyzed using HCW as a covariate. Similar treatment means between groups with the HCW adjustment indicated that the LM area response without HCW adjustment was primarily a function of body mass.

## 4. Discussion

Despite equal and adequate levels of NE_m_ and NE_g_ across treatments, MP restriction reduced the ability of RES heifers to maintain BW and BCS. In addition to BW and BCS losses, reductions in LM area based on ultrasound measurements suggest that muscle tissue was being catabolized to mobilize tissue protein in compensation for the dietary MP restriction in this study. Freetly et al. [22] reported that pregnant dams encountering a nutrient restriction may compensate for the fetus by catabolizing fat stores and lean body tissue to maintain pregnancy and normal body function.

In beef cattle, severe nutrient restriction from the last half to the last third of pregnancy appears to be required to reduce fetal growth [23]. Although the current study encompassed the majority of the second and all of the third trimester of pregnancy, the lack of birth BW response agrees with previous research indicating that the energy available to the dam may have a greater influence on birth BW than protein [24]. Since the treatments utilized in the current study were formulated to be isocaloric, it is possible that MP-restricted dams were able to overcome an MP deficiency by mobilizing body stores (particularly LM area; Table 1 and Table 2), thereby reducing the potential impacts on offspring. Dams in the current study may have been able to buffer the impacts of an MP restriction on the fetus, resulting in similar weights for calves among treatment groups at birth and weaning.

Greenwood and Cafe [25] reported that the severe growth restriction of cattle early in life resulted in reduced growth potential throughout the production cycle, although a BW equivalent to normally grown cattle could be obtained, given more time on feed. Therefore, differences in progeny BW due to maternal dietary treatment may not appear during the finishing period, given the lack of influence on birth and weaning weights.

Few studies on fetal programming have evaluated the effect of maternal protein requirements and responses to supplementation based on MP rather than dietary CP. Oversupply of MP was evaluated in late gestation, with no effect on progeny performance [13]. However, excess MP is not typically an issue in spring-calving beef cattle in forage- production systems based on low-quality forages. Gestational MP restriction in ewes during late gestation had little effect on the live performance of progeny [12]. Acton [15] also evaluated MP levels during late gestation in beef cattle and reported that the only performance response was a greater weaning weight when MP was restricted to 90% of requirements as compared to 110% of requirements. Body weight was similar among treatments for all other weight periods [15].

Beyond these MP-focused studies, there is a paucity of data in the literature where researchers have isolated the influence of protein alone by ensuring that diets are balanced to provide similar amounts of energy. A primary reason for this may be the imposition of protein restriction treatments based on dietary CP rather than MP. As a variable portion of dietary CP is ruminally degradable, which is potentially converted to microbial protein, CP does not directly translate to the protein available at the small intestine (i.e., MP). Additionally, degradable protein supports microbial synthesis, contributing to increased ruminal fermentation capacity that, in turn, typically improves energy supply to the host ruminant. Thus, CP-based treatments do not allow for the isolation of the effect of MP restriction to the ruminant. Stalker et al. [9] provided mixed-age cows with either no supplement or a 42% CP supplement at 0.45 kg/day when grazing dormant native range forage during the last trimester of gestation. Although a nutrient restriction was not imposed by Stalker et al. [9], it would be reasonable to assume that cows on the control treatment were deficient in protein during late gestation. Stalker et al. [9] did not observe differences in feedlot ADG, DMI, or feed efficiency for steer progeny due to maternal dietary treatment. Three follow-up studies with slight variations in treatment arrangements resulted in (1) no influence of maternal nutrition on heifer progeny ADG or G:F [26]; (2) a tendency for increased ADG and feed intake for steer progeny from protein-supplemented cows, but no overall difference among treatments for overall BW gain efficiency [27]; and (3) similar DMI and residual feed intake for heifer progeny from control and supplemented dams [28]. 

A two-year study was conducted, in which spring-calving cows grazed dormant forage in late gestation, with cows at one location receiving a high-nutrition treatment (0.95 kg/day of 31.6% CP supplement) and cows at a second location receiving a low-nutrition treatment (0.37 kg/day of the same supplement) delivered three times per week [10]. Although differences were detected between years, final feedlot BW, ADG, DMI, and G:F did not differ among progeny due to maternal nutritional treatment [10]. Another CP supplementation study by Banta et al. [29] provided evidence of a similar lack of response for the feedlot performance of progeny from dams fed soybean meal, soybean hull-based supplement, or whole sunflower seeds for 76 days from mid- to late-gestation. 

Summers et al. [30] compared the effects of meadow hay fed during late gestation with no supplement vs. two supplements providing 28% CP, but with differing levels of ruminally undegraded protein (59% RUP or 34% RUP). No differences were observed in ADG, reimplant or final BW, or G:F.

The results from these studies indicated little, if any, influence of CP supplementation from mid- to late-gestation on the subsequent feedlot performance of offspring. In contrast, progeny from cows grazing native range vs. improved pasture from mid- to late-gestation had reduced ADG, less total BW gain and a tendency towards decreased final BW, despite having similar initial weights upon entering the feedlot [31]. Variable performance responses observed in supplementation studies in grazing livestock are inherent due to differences in the formulation, amount, and timing of supplementation, in addition to environmental differences affecting forage quality.

Beyond these studies based on CP restriction, another approach to evaluating gestational nutrition has been to impose severe, global nutrient restriction, usually by limiting intake. Long et al. [32] fed low- (55% of NRC requirements for NE_m_ and 50% for CP) or moderate- (100% of NRC requirements) nutrition diets to cows, beginning on day 32 of gestation through day 115 of gestation, at which point cows were commingled and fed in excess of calving requirements. No differences were observed in the ADG of progeny between low- and moderate-nutrition dams. However, steers from restricted dams were heavier at the beginning of the finishing period and tended to have greater slaughter weights compared with steers from dams on the moderate-nutrition treatment, indicating that prenatal nutrition in early pregnancy had a moderate developmental programming effect on postweaning growth. A greater difference might be expected between treatments in response to the severity of the restriction.

Robinson et al. [33] conducted a stepwise regression analysis to determine the influence of maternal nutritional status during pregnancy on production characteristics up to 30 months of age. The authors reported that, although severe chronic nutritional restriction from mid-gestation through calving resulted in fetal growth retardation and a reduced BW of offspring until harvest, there were few specific effects on feed efficiency, carcass composition at similar weights, or beef quality of offspring. These results suggest that the environment and other factors affecting postnatal calf growth still play a large role in the lifetime performance of beef cattle, perhaps in conjunction with or addition to the specific nutritional environment encountered during gestation.

The impacts of maternal nutrient restriction on muscle fiber development and, ultimately, meat quality are evident based on the available literature. For example, mid-gestation restriction of CP affected muscle fiber and collagen formation in the muscle of progeny [34]. However, overall effects on carcass quality and yield were not evaluated in the cited study. Additionally, most reports evaluated a global nutrient restriction and/or utilized sheep as the experimental unit. There are limited reports of progeny carcass responses to gestational MP restriction in beef cattle. The restriction of MP during late gestation [14] was evaluated in beef cattle, with no major effect on beef quality. Gestational MP restriction in ewes during late gestation [12] did not affect the carcass traits of progeny. Acton [15] also evaluated MP levels during late gestation in beef cattle and reported the only carcass response was increased fat thickness in response to MP restricted to 90% as compared to 110% of requirements.

Pregnant ewes restricted to 50% of global nutrient requirements from day 28 to 78 of gestation resulted in the down-regulation of protein synthesis in fetal muscle, reduction in secondary myofibers, and an increase in intramuscular triglyceride content [6,35]. Fahey et al. [36] found changes in muscle characteristics of lambs born to ewes restricted to 50% of nutrient requirements from day 30 to 70 of gestation, while restriction late in gestation (day 85 to 115) reduced the weight of LM, semitendinosus, and vastus lateralis muscles of offspring. Lambs were harvested shortly after birth (14 days post-parturition) to determine muscle characteristics; therefore, longer-term impacts on muscle growth, performance, and carcass quality were not measured. 

Ford et al. [37] fed multiparous ewes at 100% or 50% of nutrient requirements between day 28 and 78 of gestation, then fed all ewes at 100% of requirements from day 79 of gestation through lambing. Lambs from nutrient-restricted ewes had an increased finished BW, greater amounts of KPH fat, and tended to have reduced LM and semitendinosus muscle weight as a percentage of HCW. The results of the above studies support the hypothesis that maternal undernutrition from early- to mid-gestation will result in increased BW and fat deposition and impact skeletal muscle development in sheep. However, the severe restrictions implemented in many of these examples may not be applicable across a wide variety of practical production situations. In addition, responses appear to be less consistent for beef cattle. For example, Greenwood et al. [23] reported significant differences in BW and growth characteristics at all stages of life (birth, weaning, backgrounding, feedlot entry, feedlot ADG, and final BW) for cattle that were severely nutrient-restricted from day 80 to 90 of gestation until birth; however, no differences in carcass composition at similar carcass weights were observed. 

Treatments for the current study were initiated during mid-gestation, with heifers either remaining on their original treatment or changing to an alternative treatment in late gestation to elucidate the effects of the timing of nutrient restriction on progeny muscle and adipose development and, ultimately, feedlot performance and carcass characteristics. Our hypothesis was that restricting MP in mid-gestation would result in reductions in skeletal muscle growth, while an MP restriction during late gestation was expected to result in increased fatness of progeny, as more mesenchymal stem cells would be expected to differentiate into adipocytes rather than muscle fibers. However, progeny from dams restricted in late gestation had an increased LM area compared with progeny from dams on the control treatment, which was unexpected. Nonetheless, this response appeared to primarily be a function of HCW, and no differences were observed for fat thickness or marbling. Similarly, Micke et al. [38] reported the LM area of both steer and heifer carcasses was increased in progeny from dams receiving a low- (70% of CP requirements) vs. high- (240% of CP requirements) nutrition diet during mid-gestation, with significant effects removed when the LM area was corrected for HCW. In contrast, Underwood et al. [31] found no differences in the LM area of steers whose dams were placed on improved pasture or native range from mid- to late-gestation; however, heavier HCW and increased 12th rib fat thickness were observed in progeny from dams grazing improved pastures. In another study, no differences in HCW, fat thickness, dressing percent, yield grade, marbling score, or LM area were observed for progeny from dams fed 100% vs. 55% of their nutrient requirements [32]. Conflicting results may be caused by altering treatment diets based on CP supply rather than MP supply.

## 5. Conclusions

This study provided evidence that MP-restricted heifers mobilized body lean tissue mass at the time the restriction was imposed, based on the observed changes in BW, BCS, and ultrasound LM area. However, MP restriction during mid- and late-gestation did not impact calf birth or weaning BW, nor did it substantially influence feedlot performance or carcass characteristics. Therefore, the hypothesis that MP restriction would result in reductions in postnatal growth and skeletal muscle, increased carcass adiposity, and reduced feed efficiency was rejected. The concept of developmental programming merits further investigation to elucidate the complex relationships between maternal nutrition, fetal development, and postnatal response. Inconsistencies in developmental programming research results may be due to the timing, intensity, and duration of nutrient restriction, influence of specific dietary restriction, and a host of genetic and environmental factors. Offspring may be buffered from the MP-level restrictions imposed in this study. Future investigation is warranted to determine the specific impacts of maternal nutrient restriction on metabolic changes and the development of specific tissues in the fetus that can impact lifetime performance and the production of beef cattle. 

## Figures and Tables

**Table 1:** Least-square means for mid-gestation treatment (CON = approximately 101% of metabolizable protein (MP) requirement supplied; RES = approximately 80% of MP requirement supplied) × time (treatment crossover and end of study) interactions for change in heifer body weight (BW), body condition score (BCS), and ultrasound measurements.

Item	Treatment Crossover		End of Study			
	CON	RES	CON	RES	SEM ^1^	*p*-Value ^2^
	Mid-gestation treatment × time interaction					
BW change, kg	−5 ^a^	−19 ^b^	21	26	5.74	0.002
BCS change	−0.30 ^c^	−0.46 ^d^	−0.18	−0.04	0.081	0.027
Longissimus muscle area change, cm^2^	−0.70 ^a^	−1.59 ^b^	−0.89	−0.58	0.273	0.042
12th rib fat thickness change, cm	0.00	−0.03	−0.08	−0.06	0.017	0.235

^1^ Standard Error of the Mean. ^2^. *p-* value for mid-gestation treatment × time interaction; ^a,b^ Within the gestation period, means lacking a common superscript differ (*p* < 0.05); ^c,d^ Within the gestation period, means lacking a common superscript tend to differ (*p* < 0.10).

**Table 2:** Least-square means for late-gestation treatment (CON = approximately 101% of metabolizable protein (MP) requirement supplied; RES = approximately 80% of MP requirement supplied) × time interactions ^1^ for change in heifer body weight (BW), body condition score (BCS), and ultrasound measurements.

Item	CON	RES	SEM ^2^	*p*-Value
BW change, kg	30 ^a^	17 ^b^	5.73	0.001
BCS change	0.00 ^a^	−0.22 ^b^	0.081	0.007
Longissimus muscle area change, cm	−0.27 ^a^	−1.20 ^b^	0.273	0.031
12th rib fat thickness change, cm	−0.06	−0.08	0.017	0.538

^1^ In Balaam’s Design, late-gestation effects on mid-gestation response would not be appropriate to consider, and thus are not presented. ^a,b^ Within the gestation period, means lacking a common superscript differ (*p* < 0.05). ^2^ Standard Error of the Mean.

**Table 3:** Main effect least square means for feedlot performance for progeny of heifers fed a control (CON = approximately 101% of metabolizable protein (MP) requirement supplied) or restricted (RES = approximately 80% of MP requirement supplied) diet during mid- and/or late-gestation ^1^.

	Mid-Gestation		Late-Gestation			*p*-Value	
Item	CON	RES	CON	RES	SEM ^2^	Mid	Late
Initial BW ^3^, kg	259	254	255	259	4.99	0.434	0.550
Final BW ^4^, kg	573	565	562	575	9.30	0.401	0.225
DMI ^5^, kg	10.06	10.06	10.06	10.06	0.143	0.984	0.972
ADG ^5^, kg	1.82	1.80	1.79	1.84	0.029	0.557	0.176
G:F ^7^	0.182	0.179	0.178	0.183	0.002	0.369	0.084

^1^ Dietary MP levels based on NRC [11] predicted requirements; mid-gestation treatment applied on mean day 148 through 216 of gestation; late-gestation treatment applied on mean day 217 of gestation through parturition; ^2^ Standard Error of the Mean; ^3^ body weight (BW) based on average of 2-day weights; ^4^ BW based on HCW/0.625 (assumed dressing percentage); ^5^ dry matter intake (DMI); ^6^ average daily gain (ADG); ^7^ Gain:feed (G:F).

**Table 4:** Main effect least-square means for carcass characteristics for progeny of heifers fed a control (CON = approximately 101% of metabolizable protein (MP) requirement supplied) or restricted (RES = approximately 80% of MP requirement supplied) diet during mid- and/or late-gestation ^1^.

Item	Mid-Gestation		Late-Gestation		SEM ^2^	*p*-Value	
	CON	RES	CON	RES		Mid	Late
HCW ^3^, kg	358	353	352	359	5.82	0.400	0.222
12th rib FT ^4^, cm	1.59	1.54	1.63	1.50	0.073	0.661	0.248
LM area ^5^, cm^2^	91.7	91.3	90.0 ^a^	92.9 ^b^	1.63	0.774	0.039
Adj LM area ^6^, cm^2^	91.3	91.7	90.6	92.3	1.88	0.756	0.231
KPH ^7^, %	2.24	2.13	2.14	2.23	0.085	0.230	0.342
Yield grade ^8^	2.76	2.67	2.79	2.65	0.135	0.597	0.443
Marbling score ^9^	514	515	520	509	22.8	0.982	0.601

^1^ Dietary MP levels based on NRC [11] predicted requirements; mid-gestation treatment applied mean day 148 through 216 of gestation; late gestation treatment applied mean day 217 of gestation through parturition; ^2^ Standard Error of the Mean; ^3^ hot carcass weight; ^4^ 12th rib fat thickness; ^5^ longissimus muscle (LM) area; ^6^ Adj. LM area determined using HCW as a covariate in the model; ^7^ kidney pelvic heart fat percentage; ^8^ evaluated according to United States Standards for Grades of Carcass Beef [19]; ^9^ 400 = Small^00^; 500 = Modest^00^; 600 = Moderate^0^; ^a,b^ within gestation period, means lacking a common superscript differ (*p* < 0.05).

## Data Availability

Data will be made available upon request to the corresponding author.
